# Identification of the Inorganic Pyrophosphate Metabolizing, ATP Substituting Pathway in Mammalian Spermatozoa

**DOI:** 10.1371/journal.pone.0034524

**Published:** 2012-04-02

**Authors:** Young-Joo Yi, Miriam Sutovsky, Chelsey Kennedy, Peter Sutovsky

**Affiliations:** 1 Division of Animal Sciences, University of Missouri-Columbia, Columbia, Missouri, United States of America; 2 Departments of Obstetrics, Gynecology and Women's Health, University of Missouri-Columbia, Columbia, Missouri, United States of America; Clermont Université, France

## Abstract

Inorganic pyrophosphate (PPi) is generated by ATP hydrolysis in the cells and also present in extracellular matrix, cartilage and bodily fluids. Fueling an alternative pathway for energy production in cells, PPi is hydrolyzed by inorganic pyrophosphatase (PPA1) in a highly exergonic reaction that can under certain conditions substitute for ATP-derived energy. Recombinant PPA1 is used for energy-regeneration in the cell-free systems used to study the zymology of ATP-dependent ubiquitin-proteasome system, including the role of sperm-borne proteasomes in mammalian fertilization. Inspired by an observation of reduced *in vitro* fertilization (IVF) rates in the presence of external, recombinant PPA1, this study reveals, for the first time, the presence of PPi, PPA1 and PPi transporter, progressive ankylosis protein ANKH in mammalian spermatozoa. Addition of PPi during porcine IVF increased fertilization rates significantly and in a dose-dependent manner. Fluorometric assay detected high levels of PPi in porcine seminal plasma, oviductal fluid and spermatozoa. Immunofluorescence detected PPA1 in the postacrosomal sheath (PAS) and connecting piece of boar spermatozoa; ANKH was present in the sperm head PAS and equatorial segment. Both ANKH and PPA1 were also detected in human and mouse spermatozoa, and in porcine spermatids. Higher proteasomal-proteolytic activity, indispensable for fertilization, was measured in spermatozoa preserved with PPi. The identification of an alternative, PPi dependent pathway for ATP production in spermatozoa elevates our understanding of sperm physiology and sets the stage for the improvement of semen extenders, storage media and IVF media for animal biotechnology and human assisted reproductive therapies.

## Introduction

Adenosine 5′-triphosphate (ATP) is a fundamental factor to maintain the life, by providing energy, and controlling the cell function and metabolism. In spermatozoa, ATP plays important roles for the movement to female reproductive tract, viability and penetration to fertilize with oocyte. Human spermatozoa treated with extracellular ATP (ATPe) have an increased motility and fertilization rates, suggesting that ATPe may be helpful to combat the male infertility [1], [2]. Addition of ATPe increased mouse fertilization rates *in vitro* without affecting the typical alterations of ATP-dependent protein tyrosine phosphorylation and acrosomal exocytosis in capacitated spermatozoa; addition of ATPe during mouse sperm capacitation altered sperm motility, and led to the elevation of intracellular calcium, which was not accompanied by hyperactivation [3]. Sperm capacitation is essential for attaining fertilizing ability. Sodium bicarbonate (NaHCO_3_), CaCl_2_ and bovine serum albumin (BSA) are required for sperm capacitation and increased protein tyrosine phosphorylation during capacitation of mouse spermatozoa [4], [5]. Therefore, ATP participated in sperm capacitation, and enhanced sperm motility during fertilization.

Prokaryotic cells do not rely solely on relatively volatile, unstable ATP as an energy source; the inorganic pyrophosphate (PPi), a stable, easy-to-store high energy compound, is able to substitute for ATP in glycolysis-related reactions under certain conditions, such as attenuated respiration [6]. PPi is a potent, mineral-binding small molecule inhibitor of crystal nucleation and growth [7], present in the extracellular matrix of most tissues and in most bodily fluids including blood [8], [9]. PPi metabolism has been studied in most detail in cultured hepatocytes and chondrocytes [10]–[15]. The intracellular PPi is generated in the mitochondria, and intra- and extracellular PPi concentrations are regulated by mitochondrial energy metabolism [10], [16]. Consequently, PPi produces mitochondrial membrane potential in an inorganic pyrophosphatase1 (PPA1)-dependent manner [17]. The ATP-derived PPi also serves as a phosphate donor in protein phosphorylation in yeast and mammalian cells [18], [19].

**Figure 1:**
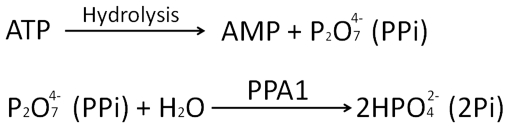
Generation of inorganic pyrophosphate (PPi). PPi is produced by the hydrolysis of ATP into AMP in cells. Inorganic pyrophosphatase (PPA1) catalyzes the hydrolysis of PPi to form 2 orthophosphates (2Pi), resulting in energy release.

Cellular PPi is yielded by various biosynthetic processes, and hydrolyzed to two inorganic phosphates (Pi) by PPA1 (Fig. 1). PPA1 is a ubiquitous metal-dependent enzyme providing a thermodynamic energy for essential biosynthetic reactions, such as DNA, RNA, protein and polysaccharide synthesis [20]–[24]. The PPA1 has been detected in bacteria [20] and yeast [21], and the soluble PPA1 was identified and characterized in *Mycoplasma suis*, which belongs to hemotrophic bacteria that attach to the surface of host erythrocytes [25]. Cellular uptake of external PPi is regulated by a membrane transporter protein, the progressive ankylosis protein (ANKH). Humans carrying a recessive mutation of ANKH gene suffer from progressive ankylosis, a joint degeneration and calcification disease caused by faulty PPi transport [26]. Unnoticed by reproductive biologists, the progressive ankylosis mice carrying a spontaneous mutation of *Ank* gene are subfertile at the young age and completely infertile beyond next reproductive cycle [26], [27].

The present study, for the first time identifies the PPi-pathway as an important component of mammalian sperm physiology. Improved boar sperm preservation and enhanced fertilization rates attained in porcine IVF model suggest that PPi might be used as a stable, inexpensive energy source to improve sperm viability during semen storage for large animal biotechnology, and as an additive to IVF media used for assisted reproductive therapy in humans.

## Results

### PPi Enhances Sperm-Zona Penetration during Fertilization and Fertilizing Ability Following Extended Storage

**Figure 2:**
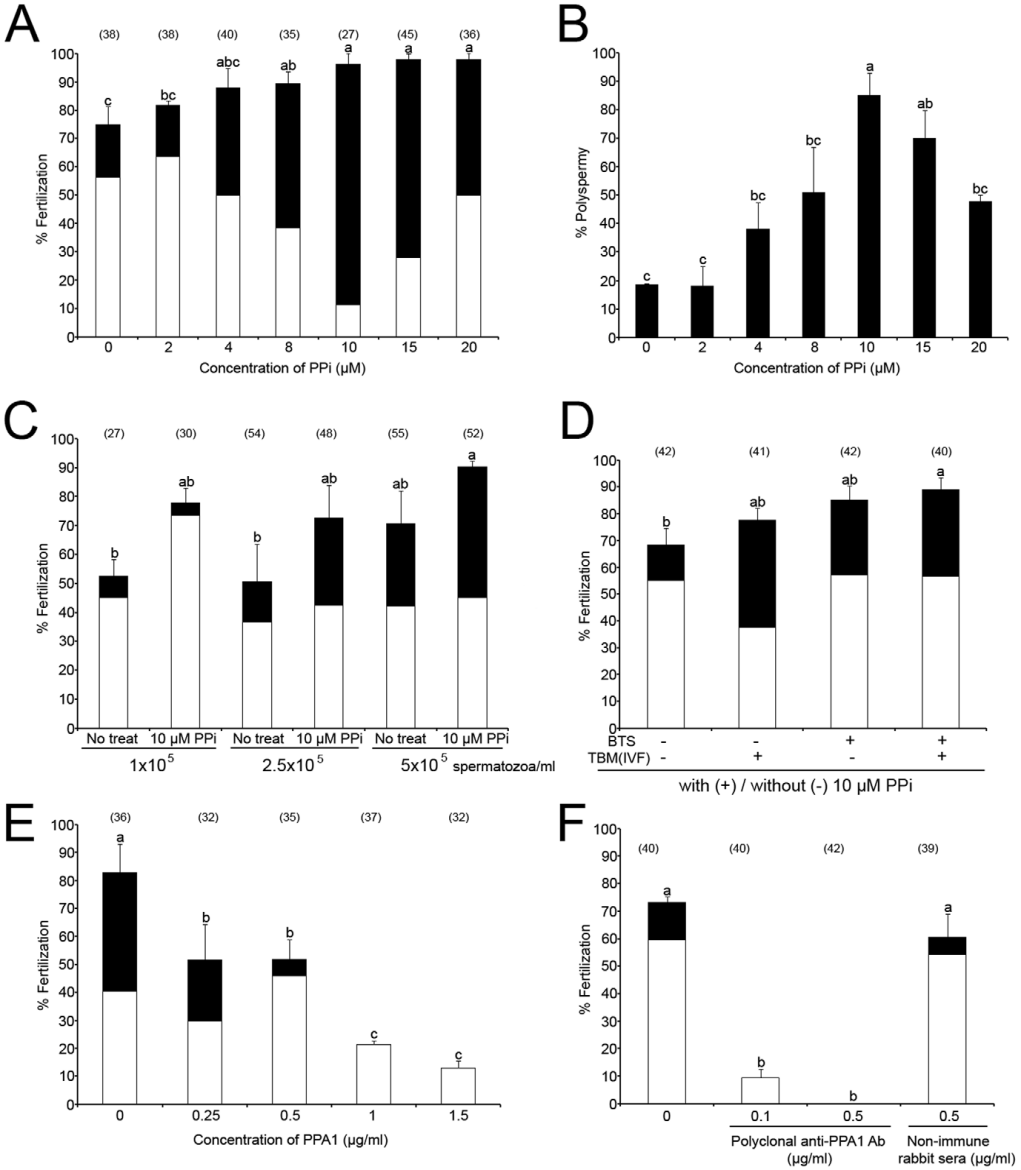
Effect of PPi on total and polyspermic fertilization during porcine IVF. Diagram indicates % monospermic (□) and % polyspermic (▪) fertilization. Values are expressed as the mean percentages of total fertilization ± SEM. Different superscripts a, b & c in each histogram denote a significant difference at p<0.05, meaning that column a is significantly different from columns b and c, column b is significantly different from columns a and c, column ab is not significantly different from either a or b, and column bc is not significantly different from columns b and c. Numbers of inseminated ova are indicated in parentheses. (A) Porcine oocytes matured *in vitro* were inseminated with a standard concentration of 1×10^6^ spermatozoa/ml, in the presence of ascending concentrations of PPi. Experiments were repeated five times. (B) The polyspermy rates, reflective of sperm fertilizing ability *in vitro* (same as panel A) dramatically increased in the presence of PPi. (C) Fertilization rates of porcine oocytes inseminated with different concentrations of spermatozoa in the presence/absence of 10 µM PPi. Experiments were repeated three times. (D) Porcine oocytes were inseminated (sperm conc. 5×10^5^ spermatozoa/ml) with (+) or without (−) 10 µM PPi in TBM, using spermatozoa stored with (+) or without (−) PPi in BTS. Experiments were repeated three times. (E) Effect of extrinsic PPA1 enzyme on porcine IVF. Oocytes were inseminated with different concentrations of purified PPA1 protein. Experiments were repeated twice. (F) Porcine oocytes were inseminated in the presence of rabbit polyclonal anti-PPA1 antibody or non-immune rabbit serum (a control of PPA1 antibody). Experiments were repeated twice.

Porcine oocytes were fertilized in the presence of PPi at different concentrations (Fig. 2A). The rates of total and polyspermic fertilization increased significantly and progressively (up to 10 µM PPi), with increasing concentrations of PPi (p<0.05). Highest polyspermy was observed after addition of 10 µM PPi (84.9% polyspermy; Fig. 2B). The mean number of spermatozoa bound to zona pellucida (ZP) decreased slightly, but not significantly with increasing concentrations of PPi (Fig. S1A). However, the percentage of acrosome-reacted spermatozoa was significantly higher in the presence of 20 µM PPi than 0–15 µM PPi (p<0.05, Fig. S1B). Since the reduction of insemination dose is desirable in the AI settings, porcine oocytes were also inseminated with reduced sperm concentrations with/without 10 µM PPi. Consistently, the percentage of total and polyspermic fertilization was augmented by PPi at 1, 2.5 and 5×10^5^ spermatozoa/ml; the increase induced by PPi was at 5×10^5^ spermatozoa/ml concentration (Fig. 2C). To determine if sperm storage in PPi-supplemented BTS extender has a beneficial effect on sperm fertilizing ability, freshly ejaculated boar spermatozoa were stored in BTS with or without 10 µM PPi for 3–4 days, and then used for IVF in the presence or absence of 10 µM PPi. The total fertilization rates of treatments with PPi in BTS or TBM were higher than a treatment without PPi, and the polyspermy was highest of all treatments with addition of PPi in BTS or TBM (IVF medium) (Fig. 2D). However, highest combined (mono+polyspermic) fertilization rate was observed with spermatozoa preserved with 10 µM PPi in BTS when used for IVF without PPi addition (Fig. 2D; third column), or with PPi in IVF medium (Fig. 2D; fourth column, p<0.05). Higher sperm motility was found in commercial BTS (BTS-IMV) with 10 µM PPi on day 6 than in all other groups (Fig. S1C). Excess PPi added into IVF medium (100–500 µM PPi) decreased fertilization rates in a dose dependent manner (Fig. S1D). In a separate trial, boar spermatozoa were stored in BTS-IMV with 10 µM PPi for 3 days, and used for IVF. One hundred percent fertilization was observed with spermatozoa preserved with 10 µM PPi, compared to 92.3% fertilization without PPi in BTS-IMV (Fig. S1E). Altogether, PPi showed a statistically significant, beneficial effect on sperm preservation and sperm fertilizing ability, suggesting that the addition of PPi in BTS might improve boar semen storage and AI.

Control experiments were conducted to deplete sperm PPi with extrinsic inorganic pyrohosphatase in form of purified PPA1. To incapacitate sperm borne PPA1, porcine oocytes were fertilized in the presence of anti-PPA1 antibody. The specificity of both reagents was established by Western blotting (see Fig. S2). Both PPA1 and anti-PPA1 antibody decreased fertilization rate in a dose dependent manner (Fig. 2E & F). No significant differences in fertilization rates were observed when the anti-PPA1 antibody was replaced with normal serum during fertilization (Fig. 2F). In addition, PPA1 was detected in the acrosome of boar spermatozoa bound to ZP when the rabbit polyclonal anti-PPA1 antibody was added in the IVF medium and detected by immunofluorescence with anti-rabbit secondary antibody after fixation of the inseminated ova (Fig. S2A). Acrosomal localization was confirmed by Western blotting of PPA1 in the isolated acrosomes (Fig. S2B). Fertilization block observed in the presence of anti-PPA1 antibody was mitigated by immunosaturation of anti-PPA1 antibody (Fig. S2C).

### PPi is Present in Porcine Seminal Plasma, Oviductal Fluid and Spermatozoa

**Figure 3:**
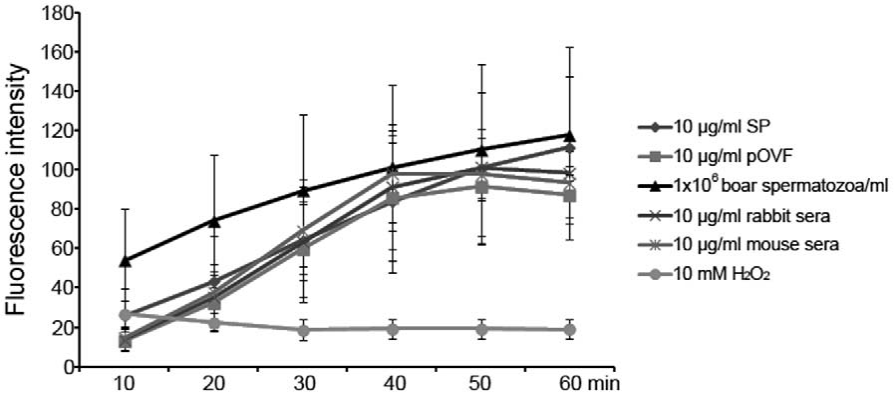
Measurement of pyrophosphate (PPi) content by fluorometric assay. PPi assay with boar seminal plasma (SP), porcine oviductal fluids (pOVF), rabbit sera, mouse sera (final conc. 10 µg/ml), boar spermatozoa (1×10^6^ spermatozoa/ml) and 10 mM H_2_O_2_ working solution (a negative control). The emitted fluorescence (no units) was measured at multiple time points to follow the kinetics of the reaction (excitation 530 nm; emission 590 nm). Experiments were repeated three times. Values are expressed as the mean of fluorescence intensity ± SEM.

The content of PPi was measured in boar seminal plasma (SP), porcine oviductal fluids (pOVF) and boar spermatozoa by fluorometric assay. The fluorescence intensities increased in pOVF, SP, spermatozoa, mouse sera and rabbit sera. The boar spermatozoa, mouse sera, and rabbit sera showed higher fluorescence intensities than SP or pOVF at 40 min of acquisition (98.2–101.1 vs. 83.8 & 85.5; Fig. 3). However, the intensities of pOVF, mouse sera and rabbit sera decreased gradually after 40 min. Only the SP and spermatozoa showed continuous increase of fluorescence intensity during measurement (fluorescence intensities: 111.5 & 117.7 at 60 min, p<0.05). A negative control, 10 mM H_2_O_2_, showed low fluorescence intensities during measurement, most likely due to bleaching of fluorescence (Fig. 3). As an additional negative control, PPi was measured in the presence of glucose, which reportedly produces aberrant results with PPi kit, which was a concern with regard to high PPi levels observed in SP and pOVF. However, in our study, addition of glucose produced a decreasing not an increase in measured PPi levels (Fig. S3).

### Detection and Localization of PPA1 in Porcine Seminal Plasma, Oviductal Fluid and Spermatozoa

**Figure 4:**
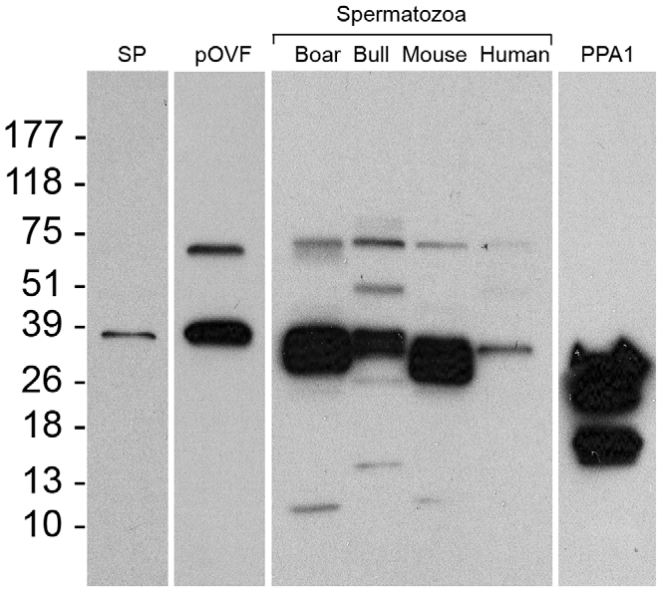
Detection of inorganic pyrophosphatase (PPA1) by Western blotting. Boar seminal plasma (SP; 20 µg/ml), porcine oviductal fluid (pOVF; 100 µg/ml), and boar, bull, mouse and human spermatozoa (all at 1×10^6^ spermatozoa/ml) were extracted to perform the protein analysis. Equal protein loads were used. Distinct band at ∼32 kDa was detected by rabbit polyclonal anti-PPA1 antibody. The purified PPA1 (extreme right lane; 1 µg/ml; Sigma I1643) from *S. cerevisiae* was used as a control protein.

**Figure 5:**
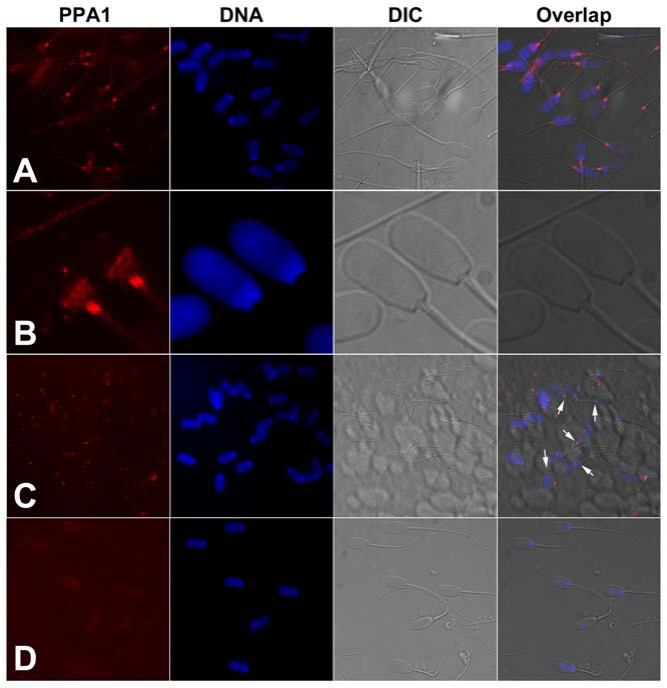
Localization of inorganic pyrophosphatase (PPA1; red) in spermatozoa by immunofluorescence. (A, B) Whole mount immunofluorescence of boar spermatozoa. Most prominent labeling is observed in the sperm tail connecting piece and in the postacrosomal sheath of the sperm head. (C) Identical labeling (arrows) is observed in spermatozoa attached to oocyte zona pellucida at 30 min after gamete mixing during IVF. (D) Negative control with anti-PPA1 antibody immunosaturated with full length PPA1 protein. DNA was counterstained with DAPI (blue). Epifluorescence micrographs were overlapped with parfocal transmitted light photographs acquired with DIC optics.

Protein band corresponding to the calculated mass of PPA1 (32 kDa) was detected in boar seminal plasma, in porcine oviductal fluid, and in boar, bull, mouse and human spermatozoa by Western blotting with rabbit polyclonal anti-PPA1 antibody (Fig. 4). Minor bands of higher (∼51 and 75 kDa) or lower mass (∼13 kDa in boar and ∼18 kDa in bull) were observed in each sperm sample, likely corresponding to posttranslational protein modification and degradation products of PPA1. The purified PPA1 from baker's yeast (*S. cerevisiae*), used as a positive control protein, also showed the expected band at 32 kDa and the minor bands between 15 to 26 kDa (Fig. 4). As a control, the membrane was incubated with immunosaturated anti-PPA1 antibody and GAR-IgG-HRP, and showed no specific bands (Fig. S4). Immunofluorescence detected a prominent labeling of PPA1 in the sperm tail connecting piece and in the postacrosomal sheath of boar spermatozoa (Fig. 5A&B). Identical labeling was found in the connecting piece of spermatozoa attached to oocyte zona pellucida at 30 min of IVF (arrows, Fig. 5C). Negative control with anti-PPA1 antibody immunosaturated with full length recombinant PPA1 protein showed no such fluorescence (Fig. 5D), and neither did labeling of non-permeabilized spermatozoa (Fig. S5).

### Supplementation with PPi Improves Sperm Viability during Semen Preservation

**Figure 6:**
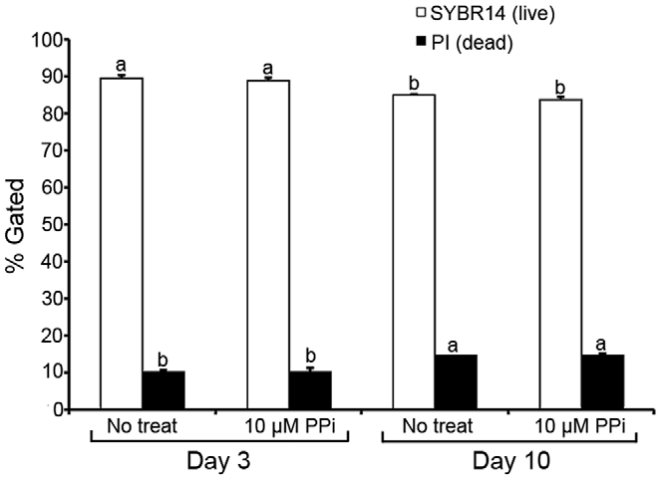
Sperm viability during sperm storage with/without PPi. Percentages of viable spermatozoa are based on dual SYBR14 (live sperm) and PI (dead sperm) labeling. Experiments were repeated three times. Values are expressed as the mean percentages ± SEM. Different superscripts a & b in histogram denote significant differences at p<0.05, meaning that column a is significantly different from column b.

Following an industry practice for boar semen storage, fresh boar semen was diluted in BTS extender and stored at room temperature (15–17°C) for 10 days [28]. The base extender is designed for short term storage (3–5 days); however, we prolonged the storage period up to 10 days to compare sperm viability and mitochondrial membrane potential between storage days 3 and 10 in the presence/absence of 10 µM PPi. Sperm viability was assessed by flow cytometry using SYBR14/PI viability kit. Mitopotential was measured with JC-1 dye. Supplementation with PPi altered the histograms and scatter diagrams of fluorescence produced by the above probes (Fig. S6A–D); a vehicle control, DMSO produced no fluorescence (Fig. S6E). The percentage of live spermatozoa was higher on day 3 than on day 10 (p<0.05), but there was no significant difference between control spermatozoa and those supplemented with 10 µM PPi (Fig. 6). Contrary to viability, PPi supplementation augmented the content of metabolically active spermatozoa with polarized mitochondrial membranes on day 3 (Fig. S7). Similar tendency was observed in spermatozoa preserved with 10 µM PPi for 10 days (Fig. S7). Interestingly, a slight, but statistically significant reduction in sperm ATP content was observed in fresh and stored semen in the presence of PPi (Fig. S8).

### PPi Supplementation Increases Sperm Proteasomal-Proteolytic Activity

**Figure 7:**
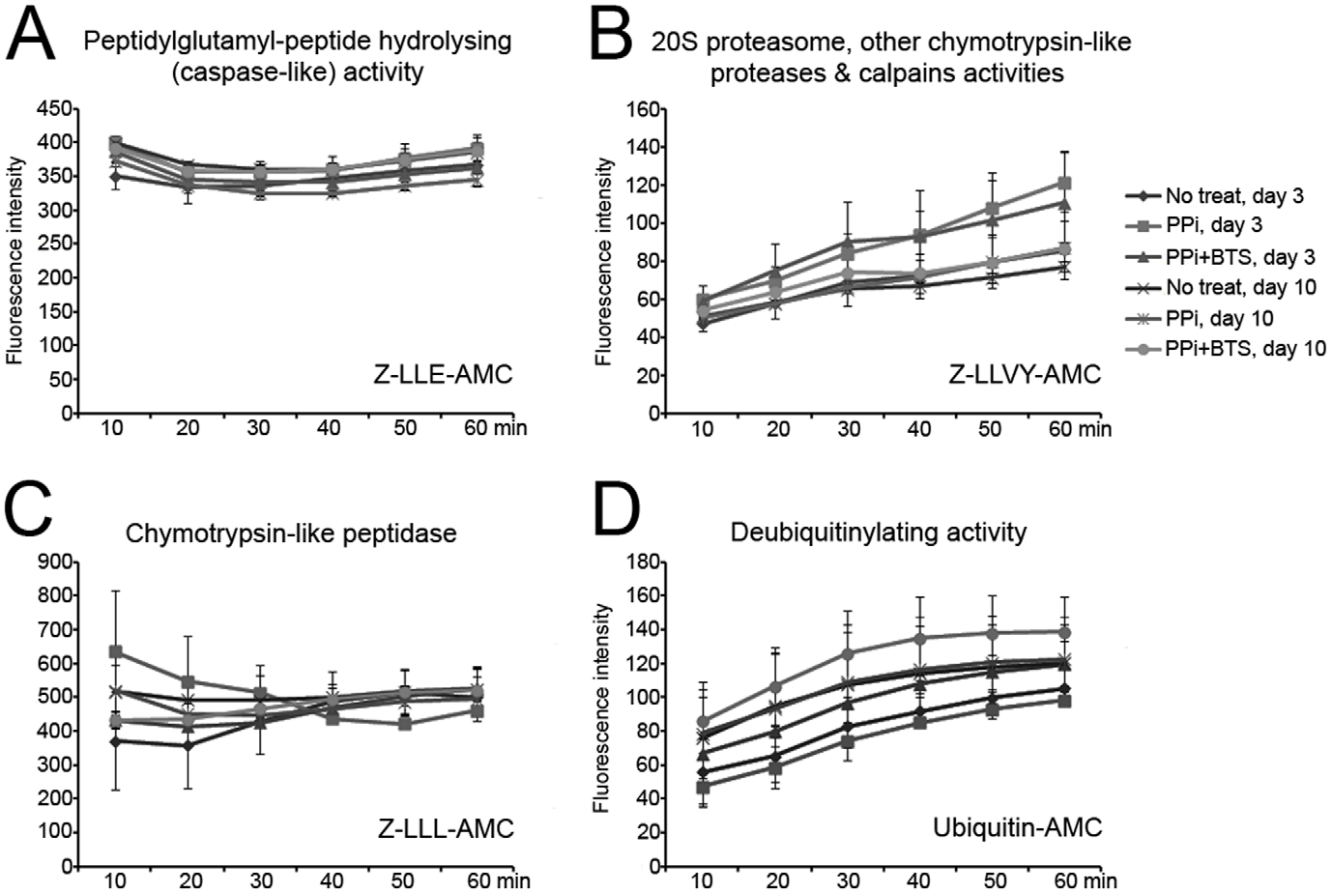
Effect of PPi on proteasomal enzymatic activities of stored boar spermatozoa. Fresh boar spermatozoa were stored in BTS with/without 10 µM PPi for 3 or 10 days (No treat/PPi+BTS). Proteasomal proteolytic and deubiquitinating activities were measured using specific fluorometric substrates Z-LLE-AMC (A), Z-LLVY-AMC (B), Z-LLL-AMC (C) and ubiquitin-AMC (D). In a separate treatment group, PPi (10 µM) was added before measurement to spermatozoa preserved without PPi in BTS (PPi). The emitted fluorescence (no units) was measured at multiple time points to follow the kinetics of the reaction (Excitation 380 nm; emission 460 nm). Experiments were repeated three times. Values are expressed as the mean of fluorescence intensity ± SEM.

The proteasomal-proteolytic and deubiquitinating activities, which are essential for fertilization, were assayed using specific fluorometric substrates Z-LLL-AMC, Z-LLVY-AMC, Z-LLE-AMC and ubiquitin-AMCs in spermatozoa stored for 3 and 10 days, with or without PPi (“PPi+BTS” treatment; Fig. 7). Alternatively, 10 µM PPi was added to semen preserved without PPi at the time of assay (“PPi” treatment). Higher chymotrypsin-like PGPH activity (Z-LLE-AMC substrate) was measured in PPi treatment on day 3 (relative fluorescence of 392.1; no units) and PPi+BTS on day 10 (relative fluorescence of 388), compared to other treatments at 60 min (363.1–386.1; Fig. 7A, p<0.05). Chymotrypsin-like proteasomal core activity (Z-LLVY-AMC substrate) gradually increased during measurement in all groups (Fig. 7B). The PPi+BTS and PPi treatments showed higher fluorescence intensities with this substrate, compared to controls at 60 min (110.8–121.5 vs. 85.7; Fig. 7B, p<0.05). The highest fluorescence intensity was observed in PPi at 10 min, but the intensity decreased progressively during measurement (Fig. 7C). Chymotrypsin-like activity showed no differences between treatments (Fig. 7C). On the contrary, a higher deubiquitinating activity (ubiquitin-AMC) was observed in PPi+BTS treatment on day 10, compared to other treatments (relative fluorescence of 138.9 vs. 98.4–122.7; Fig. 7D). Overall, supplementation with PPi increased the proteasomal-proteolytic and deubiquitinating activities in spermatozoa and showed beneficial effect during sperm preservation.

### PPi Transporter ANKH is Present in Boar Spermatozoa

**Figure 8:**
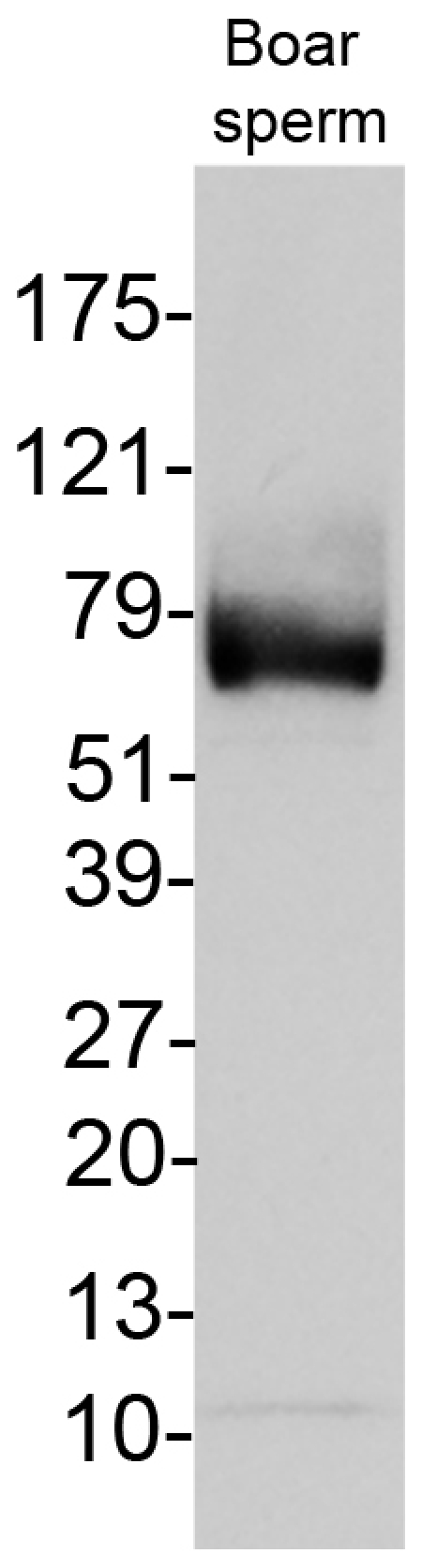
Western blotting of PPi transporter protein ANKH in boar spermatozoa (anticipated mass: 76 kD).

**Figure 9:**
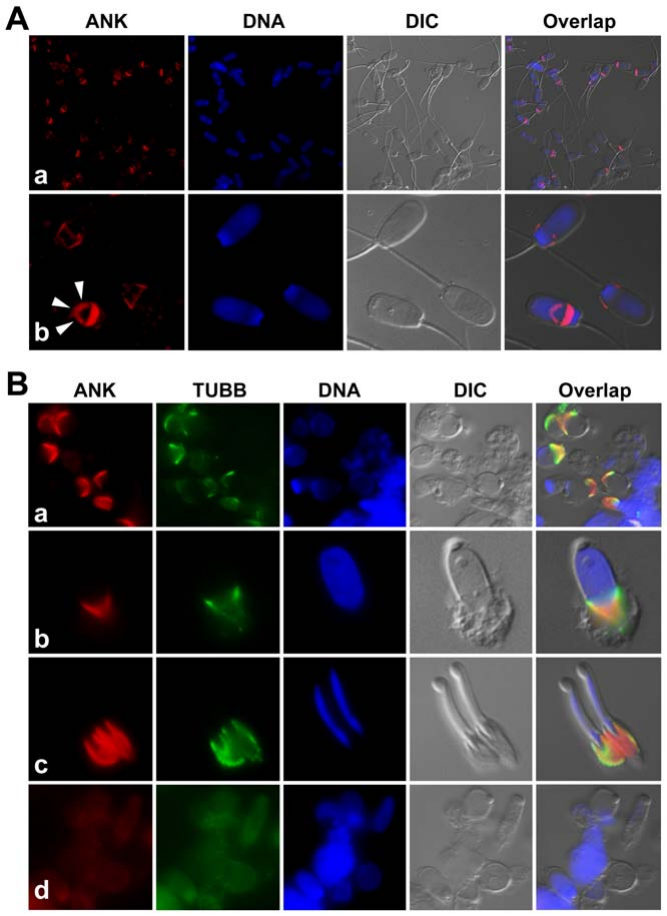
Immunofluorescence of ANKH (red) in boar spermatozoa (A) and spermatids (B). (A) Prominent labeling is present in the postacrosomal sheath, and in the equatorial segment (arrowheads in panel b) of ejaculated spermatozoa. (B) During spermiogenesis, ANKH colocalizes with tubulin (TUBB, green) in the microtubules of the spermatid caudal manchette (a–c) at all steps of spermatid elongation during which the manchette is present (late round/early elongating spermatid-a; late elongating spermatid-b & c; top and side view-d, respectively). This pattern is typical of the deposition of proteins into postacrosomal sheath that serve a structural or metabolic/signaling purpose in fully differentiated spermatozoa. Microtubules were labeled with monoclonal antibody against tubulin beta (TUBB). DNA was counterstained with DAPI (blue). Epifluorescence micrographs were overlaid with parfocal transmitted light photos acquired with DIC optics.

The progressive ankylosis protein (mouse Ank and the human homolog, ANKH), is a pyrophosphate transporter that regulates PPi transport across plasma membrane between intra- and extracellular compartments [26], [29]. Reduced fertility has been reported in *ank* mutant mice [26]. Western blotting detected ANKH band of anticipated mass (76 kD) in boar sperm extracts (Fig. 8). Immunofluorescence detected ANKH in the post acrosomal sheath (PAS) and equatorial segment of boar spermatozoa, and in the association with caudal manchette in elongating spermatids, a pattern typical for PAS proteins deposited during late steps of spermiogenesis (Fig. 9).

## Discussion

The present study, for the first time, shows the presence of PPi and the elements of PPi metabolism pathway in spermatozoa of a mammal. Inorganic pyrophosphate is a product of biosynthetic reactions that utilize ATP; it is hydrolyzed by inorganic pyrophosphatase PPA1, an important enzyme for energy metabolism [20], [21]. PPA1 has been implicated in the regulation of metabolism, growth and development in plants [22], and even in the development and molting in the parasitic roundworm *Ascaris* [30]. During cell division of *S. cerevisiae*, PPA1 is essential for mitochondria genome replication [31]. PPi (P_2_O_7_^4−^) is formed by the hydrolysis of ATP into AMP in cells, then, converted by PPA1 into two molecules of inorganic orthophosphate (Pi). Besides participating in the regulation of intracellular functions and extracellular crystal deposition, PPi serves as a primordial “high energy” source under certain conditions [19]. One such instance could be in spermatozoa after ejaculation; sperm transport through oviduct and sperm penetration through egg vestments requires high energy to fuel sperm flagellar movement. Coinciding with this possible function, PPA1 is detectable in the sperm tail connecting piece, harboring sperm centriole and anchoring flagellar outer dense fibers and microtubule doublets. In addition, both PPA1 and ANKH are concentrated in the sperm head postacrosomal sheath, with ANKH also detected in the equatorial segment, overlapping with the distal part of sperm acrosome. From these locations, the PPi-metabolizing pathway could convey energy for flagellar movement and for acrosomal function during sperm-zona penetration. In this regard, we have observed an increase in proteasomal enzymatic activities, essential for sperm-zona interactions (reviewed by [32]), stimulated by PPi supplementation *in vitro*. Finally, the PPi pathway in the sperm head and flagellum could support protein phosphorylation during sperm capacitation that is observed both *in vitro* and *in vivo*, in the oviductal sperm reservoir. Altogether, present studies suggest the ability of mammalian spermatozoa to utilize PPi as an energy source. Thus, high concentration of PPi in the oviductal fluid may serve an exact purpose during sperm transport in female reproductive system. This may be unique to male gametes, since we have not observed any beneficial effect of PPi supplementation on oocyte maturation and preimplantation embryo development *in vitro*, at least not at the concentrations that benefit sperm viability (data not shown).

The concentration of cytosolic PPi is precisely regulated in mammalian cells [33], [34], and tissue specific differences in PPA1 activity have been reported in mammals [35], [36]. In this study, the presence of PPi was confirmed in boar seminal plasma (SP) and porcine oviductal fluids (pOVF) by established fluorometric method (Fig. 3) [37], [38]. Therefore, PPi could be utilized as an energy source during sperm transport and sperm-egg interactions, as the spermatozoa undergo capacitation, acrosome reaction and sperm-zona penetration. Sperm motility, capacitation and acrosome reaction all occur during porcine IVF, which, as we show, benefits from supplementation with PPi, and is inhibited by extrinsic, purified PPA1 and by anti-PPA1 antibodies. Such a block is possible because events regulated by ATP dependent activities, such as proteasomal proteolysis, occur on the cell surface in fertilizing spermatozoa. Previously, PPA1 has been implicated in the bacterial energy metabolism [20], [21], and the activity of recombinant *Mycoplasma suis* PPA1 increased with increasing concentrations of PPi [25]. Moreover, the addition of PPi increased the accumulation of the permeant cation tetraphenylphosphonium (TTP^+^) in yeast mitochondria, where PPi has a role of energy donor [17].

In the present study, PPi was added in sperm extender or IVF medium to enhance sperm viability during sperm storage and sperm penetration during fertilization. Extender studies have practical implications for large animal biotechnology and particularly for artificial insemination in pigs, where fresh, as opposed to cryopreserved semen is distributed, necessitating handling and long term storage (up to 7 days) at room temperature. Mimicking standard procedures of swine industry [28], freshly ejaculated boar spermatozoa were diluted in BTS, a common industrial semen extender used for a short-term storage of boar semen (3–5 days at 15–17°C). However, we prolonged the storage periods up to 10 days at a slightly higher temperature (20–22°C), to compare sperm viability and mitochondrial membrane potential in the presence/absence of 10 µM PPi. Sperm viabilities and mitopotential were assessed by flow cytometry using commercial SYBR14/PI and JC-1 kits. The SYBR14/PI kit discriminates between live and dead sperm spermatozoa [39]. The JC-1 kit measures the mitochondrial membrane potential by labeling metabolically active mitochondria in the sperm tail midpiece, which provide energy for sperm movement and sperm-zona penetration during fertilization. Higher percentages of polarized, live spermatozoa were observed in semen preserved with 10 µM PPi at day 3 and day 10 of storage. Thus, PPi improved sperm survival and sustained mitopotential during extended semen storage. Sperm viability decreases during storage, and reduces sperm fertilizing ability due to the loss of ATP and cAMP, and reduced calcium uptake [40]. This could be a result of accelerated sperm metabolism and ATP utilization, or reduced sperm ATP output in the presence of an alternative energy source in form of PPi.

High polyspermy is common in porcine oocytes matured *in vitro*, compared to *in vivo* oocytes, caused by delayed and incomplete cortical granule (CG) exocytosis [41], [42]. It is well known that the glycoproteins from oviductal fluid play an important role in preventing polyspermy in the domestic pig [43], [44]. Such glycoproteins are not present on the surface of oocytes retrieved from the ovary for *in vitro* maturation. In the present study, we used an optimized IVF system to reduce polyspermy and maintained the average monospermic fertilization rates between 50 to 60%. However, when PPi was added into IVF medium, especially at 10 µM concentration, polyspermy was accelerated, reflecting an overall increase in fertilizing ability of PPi-exposed spermatozoa. Such an increasing of polyspermy would not be a concern following artificial insemination, since the oocytes inside female reproductive tract are protected from polyspermy by oviductal glycoproteins. In order to reduce the loss of fertilizing ability of stored semen in artificial insemination (AI) trials, researcher doubled the usual dose of AI-sperm (3×10^9^) on day 4 of storage, and used boars with best proven AI-fertility [28]. When the number of spermatozoa in the inseminate was reduced to 0.5×10^9^, a significant reduction of fertility from 90.2% to 58.3% was observed [28]. Sperm number per AI-dose has a direct effect on the profits in AI-industry. Thus, an additive that would allow for reduced AI dose in either fresh or stored boar semen would have a significant economic impact. There is now two-fold evidence suggesting that PPi, an inexpensive, relatively stable compound could be such an additive. In addition to improving sperm viability during storage, addition of PPi improved the fertilizing ability of both fresh and stored spermatozoa in our IVF trials. Building on these *in vitro* results, the use of PPi as a sperm booster in commercial AI is currently under investigation.

Enzymatic activities of sperm borne proteasomes are measurable by standard fluorometric substrate assays and can be used to gauge fertilizing potential [45]. Sperm proteasomes are essential for sperm-zona penetration during porcine fertilization *in vitro* [46], [47]. The proteasome is composed of one or two 19 S regulatory complexes capping the 20 S proteasomal core. ATP is essential for the function and structural integrity of the 19 S complex, involved in the recognition and priming of ubiquitinated proteins destined for proteasomal degradation; it is not required for the function of 20 S core, where substrate proteolysis occurs. ATP is also required for enzymatic reactions during protein ubiquitination, which may occur in spermatozoa during fertilization [48]. Often, when such reactions are reconstituted in cell-free systems using cell extracts that likely contain PPi, PPA1 is added as energy booster. In fact, the present study was inspired by our previous studies, in which boar sperm acrosomal extracts were used to demonstrate their intrinsic ubiquitin-activating and conjugating activities [48]. Unique to spermatozoa, the ubiquitin-proteasome system involved in fertilization is extracellular and thus sensitive to large molecules that can only act intracellularly in other cell types. In the present study, significantly higher proteasomal-proteolytic and deubiquitinating activities were measured in spermatozoa preserved with PPi or after PPi addition during measurement. This indicates that PPi enhanced sperm proteasome activity, providing a possible explanation for benefits of PPi supplementation during fertilization.

The PPi transporter ANKH plays an important role in the regulation of PPi levels in extracellular space and cytoplasm. The role of ANKH has been defined based on a spontaneous mutation of *Ank* gene in a mouse model of arthritis/progressive ankylosis [26]. These *ank* mutant mice showed malformations such as arthropathy and intrarenal calcification. ANKH has also been detected in neuronal cell, and implicated in regulation of ATP levels and PPi release [49]. Additionally, ANKH is expressed in murine kidney and bone cells, where it facilitates pyrophosphate transport [50]. In the present study, we detected ANKH in boar spermatozoa by Western blotting and localized to acrosome-covered equatorial segment and postacrosomal sheath by immunofluorescence. During spermiogenesis, ANKH associated with caudal manchette, a pattern typical of late-translated PAS proteins such as postacrosomal sheath WW domain-binding protein (PAWP/WBP2NL) [51]. Additionally, PPA1 has been detected in the sperm tail connecting piece, in the post-acrosomal sheath and in the acrosome of boar spermatozoa by immunofluorescence. It remains to be determined if ANKH in sperm head regulates PPi import or export, or both. Also, current methodology does not allow for assessing compartmentalization of PPi, which would be expected to be present in the sperm head acrosome and sperm tail principal piece in addition to PAS and sperm tail connecting piece where PPA1 and ANKH concentrate. Reduced sperm motility and number of spermatozoa with normal morphology were reported in patients with ankylosis spondylitis, compared to healthy men [52], suggesting that ANKH may be involved in the regulation of spermatogenesis and/or sperm motility.

In conclusion, the addition of PPi in IVF medium enhanced sperm penetration during porcine fertilization, increased sperm proteasomal enzymatic activities, and improved sperm viability during extended semen storage. Such data suggest that PPi would be a beneficial component to improve semen extenders, storage media and IVF media for animal biotechnology and human assisted reproductive therapies.

## Materials and Methods

### Semen Collection and Processing

All studies involving vertebrate animals were completed under the strict guidance of ACUC protocol number #A3394-01, approved by the Animal Care and Use Committee of the University of Missouri. Semen was collected from proven fertile adult Duroc boars 15–22 months of age. The boars were placed on a routine collection schedule of one collection per week. The sperm-rich fraction of ejaculate was collected into an insulated vacuum bottle. The sperm-rich fractions of ejaculates with greater than 85% motile spermatozoa were used. Semen volumes were determined with a graduated cylinder. Sperm concentrations were estimated by a hemacytometer (Fisher Scientific, Houston, TX). The percentage of motile spermatozoa was estimated at 38.5°C by light microscope at 250×. Semen was slowly cooled to room temperature (20–23°C) by 2 h after collection. Semen was diluted with Beltsville thawing solution (BTS; 3.71 g glucose, 0.60 g trisodium citrate, 1.25 g ethylenediamine tetraacetic acid, 1.25 g sodium bicarbonate, 0.75 g potassium chloride, 0.06 g penicillin G and 0.10 g streptomycin in 100.0 ml distilled water) [53] diluent to a final concentration of 35×10^6^ spermatozoa/ml in 100 ml of BTS diluent. The diluted semen was stored in styrofoam box at room temperature for 10 days. Unless otherwise noted, all chemicals used in this study were purchased from Sigma Chemical Co. (St. Louis, MO).

### Collection and *In Vitro* Maturation (IVM) of Porcine Oocyte

Ovaries were collected from prepubertal gilts at a local slaughterhouse and transported to the laboratory in a warm box (25–30°C). Cumulus oocyte complexes (COCs) were aspirated from antral follicles (3–6 mm in diameter), washed three times in HEPES-buffered Tyrode lactate (TL-HEPES-PVA) medium containing 0.01% (w/v) polyvinyl alcohol (PVA), then washed three times with the maturation medium [54]. Each time, a total of 50 COCs were transferred to 500 µl of the maturation medium that had been covered with mineral oil in a 4-well multidish (Nunc, Roskilde, Denmark) and equilibrated at 38.5°C, with 5% CO_2_ in the air. The medium used for oocyte maturation was tissue culture medium (TCM) 199 (Gibco, Grand Island, NY) supplemented with 0.1% PVA, 3.05 mM D-glucose, 0.91 mM sodium pyruvate, 0.57 mM cysteine, 0.5 µg/ml LH (L5269, Sigma), 0.5 µg/ml FSH (F2293, Sigma), 10 ng/ml epidermal growth factor (E4127, Sigma), 10% porcine follicular fluid, 75 µg/ml penicillin G, and 50 µg/ml streptomycin. After 22 h of culture, the oocytes were washed twice and cultured in TCM199 without LH and FSH for 22 h at 38.5°C, 5% CO_2_ in air.

### *In Vitro* Fertilization (IVF) and Culture of Porcine Oocyte

After the completion of oocyte maturation, cumulus cells were removed with 0.1% hyaluronidase in TL-HEPES-PVA medium and were washed three times with TL-HEPES-PVA medium and Tris-buffered (TBM) medium [54] containing 0.2% BSA (A7888, Sigma), respectively. Thereafter, 25–30 oocytes were placed into each of four 50 µl drops of the TBM medium, which had been covered with mineral oil in a 35 mm polystyrene culture dish. The dishes were allowed to equilibrate in the incubator for 30 min until spermatozoa were added for fertilization. One ml of liquid semen preserved in BTS diluent was washed twice in PBS containing 0.1% PVA (PBS-PVA) at 800×*g* for 5 min. At the end of the washing procedure, the spermatozoa were resuspended in TBM medium. After appropriate dilution, 50 µl of this sperm suspension was added to 50 µl of the medium that contained oocytes to give a final sperm concentrations of 1–10×10^5^ spermatozoa/ml. Different concentrations of inorganic pyrophosphate (PPi; S6422, Sigma) were added to fertilization drops (final concentrations; 0–20 µM) at the time of sperm addition. Oocytes were co-incubated with spermatozoa for 6 h at 38.5°C, 5% CO_2_ in air. At 6 h after IVF, oocytes were transferred into 100 µl NCSU23 containing 0.4% BSA (A6003, Sigma) for further culture during 16–20 h.

### Immunofluorescence and Evaluation of Fertilization Rates

Spermatozoa/oocytes were fixed in 2% formaldehyde for 40 min at room temperature, washed, permeabilized in PBS with 0.1% Triton-X-100 (PBS-TX) and blocked for 25 min in PBS-TX containing 5% normal goat serum. Spermatozoa/oocytes were incubated with rabbit polyclonal anti-pyrophosphatase 1 (PPA1) antibody (1∶200 dilution; #ab96099, Abcam, San Francisco, CA) or rabbit polyclonal anti-ANKH antibody (1∶200 dilution; #SAB1102581, Sigma) for 40 min, then incubated with goat-anti-rabbit (GAR)-IgG-TRITC (1/80 dilution; Zymed Inc., San Francisco, CA). For the evaluation of fertilization, oocytes/zygotes were fixed with 2% formaldehyde for 40 min at room temperature, washed three times with PBS, permeabilized with PBS-TX for 40 min at room temperature, and stained with 2.5 µg/ml DAPI (Molecular Probes, Eugene, OR) for 40 min. Oocytes with two or more pronuclei and at least one sperm tail in the ooplasm were recorded as fertilized. In order to count the number of spermatozoa bound to zona pellucida (ZP) or acrosome reacted spermatozoa, oocyte were fixed and stained with DAPI and acrosome-binding lectin PNA-FITC (Molecular Probes) after IVF 30 min (5×10^5^ spermatozoa/ml). Image acquisition was performed on a Nikon Eclipse 800 microscope (Nikon Instruments Inc., Melville, NY) with Cool Snap camera (Roper Scientific, Tucson, AZ) and MetaMorph software (Universal Imaging Corp., Downington, PA).

### Western Blotting

For Western blotting [46], extracts of 1×10^6^ spermatozoa/ml were loaded per lane on 4–20% PAGEr® gels (Lonza Rockland), then transferred, blocked and probed with anti-PPA1 or anti-ANKH antibodies (1∶2,000 dilution) overnight. The membranes were then incubated with the HRP-conjugated goat anti-rabbit IgG (GAR-IgG-HRP), reacted with chemiluminiscent substrate (SuperSignal, Pierce) and visualized by exposing to Kodak BioMax Light film.

### Pyrophosphate Assay

The measurement of pyrophosphate (PPi) was performed using PiPer™ Pyrophosphate Assay Kit (Cat. No. P22062, Molecular Probes), following manufacturer's protocol. The samples were prepared using 1× reaction buffer (Kit) with boar seminal plasma (SP), porcine oviductal fluids (OVF), rabbit sera, mouse sera (final conc. 10 µg/ml), boar spermatozoa (1×10^6^ spermatozoa/ml) and 10 mM H_2_O_2_ working solution (a negative control). Fifty µl samples were loaded into black 96-well (Costar-Corning, Corning, NY), and then 50 µl working solutions were added into sample, respectively. The emitted fluorescence (relative values; no units) was measured every 10 min for a period of 1 h, yielding a curve of relative fluorescence (excitation: 530 nm, emission: 590, Thermo Fluoroskan, ThermoFisher Scientific).

### Flow Cytometric Analysis of Sperm Viability and Mitochondrial Membrane Potential

Boar spermatozoa were washed twice with PBS-PVA, and sperm concentration was adjusted to 1×10^6^ spermatozoa/ml in PBS-PVA. The sperm viability was assessed by LIVE/DEAD® Sperm Viability Kit (L-7011, Molecular Probes) which contains DNA dyes SYBR14 and propidium iodide (PI), following manufacturer's protocol. Sperm samples (198 µl) were loaded onto a 96-well plate. SYBR14 (1 µl; final conc. 100 nM) and PI (1 µl; final conc. 12 µM) were added to sperm samples and incubated for 10 min at 37.5°C in dark. Flow cytometric analysis was performed using a Guava EasyCyte™ Plus flow cytometer (Guava Technologies, IMV Technologies, L'Aigle, France). For each samples, 5,000 events were analyzed by Guava ExpressPro Assay program, using standard manufacturer's settings. For assessment of sperm mitopotential, boar spermatozoa were stained with JC-1 (Cat. No. 4500-0250, MitoPotential Kit, IMV), and measured using manufacturer's settings. For negative controls, DMSO or no staining solution were added to sperm samples.

### Measurement of Proteasomal-Proteolytic Activity

Sperm preserved in BTS with/without 10 µM PPi were loaded into a 96-well black plate (final sperm conc. 1×10^6^ spermatozoa/ml), and incubated at 37.5°C with Z-LLE-AMC (a specific substrate for 20 S chymotrypsin-like peptidyl-glutamylpeptide hydrolyzing [PGPH] activity not sensitive to MG132; final conc. 100 µM; Enzo Life Sciences, Plymouth, PA), Z-LLVY-AMC (a specific substrate for 20 S proteasome and other chymotrypsin-like proteases, as well as calpains; final conc. 100 µM; Enzo), Z-LLL-AMC (a specific substrate for 20 S chymotrypsin-like activity sensitive to proteasomal inhibitor MG132; final conc. 100 µM; BostonBiochem, Cambridge, MA) or ubiquitin-AMC (specific substrate for ubiquitin-C-terminal hydrolase activity; final conc. 1 µM; Enzo) for 1 h. The emitted fluorescence (no units) was measured every 10 min for a period of 1 h, yielding a curve of relative fluorescence (excitation: 380 nm, emission: 460, Thermo Fluoroskan, ThermoFisher Scientific).

### Statistical Analysis

Analyses of variance (ANOVA) were carried out using the SAS package in a completely randomized design. Duncan's multiple range test was used to compare values of individual treatment when the F-value was significant (p<0.05).

## Supporting Information

Figure S1 **(A) Effect of PPi on sperm-zona binding.** Porcine oocytes were inseminated (sperm conc. 5×10^5^ spermatozoa/ml) with various concentrations of PPi for 30 min, fixed and stained with DNA stain DAPI. The numbers of spermatozoa bound per zona-pellucida (ZP) were counted under epifluorescence microscope. Values are expressed as the mean number ± SEM. Different superscripts a, b & c in each histogram denote a significant difference at p<0.05, meaning that column a is significantly different from columns b and c, column b is significantly different from columns a and c, column ab is not significantly different from either a or b, and column bc is not significantly different from columns b and c. Numbers of inseminated ova are indicated in parentheses. (B) The percentage of acrosome-reacted spermatozoa of panel A (PNA-FITC stained). Values are expressed as the mean percentages ± SEM. Different superscripts a & b in each group of columns denote a significant difference at p<0.05. (C) Effect of PPi supplementation on the viability of spermatozoa stored in commercial and custom made BTS extenders. Boar spermatozoa were preserved in BTS-IMV (IMV technologies, France) or BTS-HM (homemade) with/without 10 µM PPi for 7 days at room temperature. The percentage of motile spermatozoa was estimated at 38.5°C using a light microscope at 250× magnification. Higher sperm motility was observed in BTS-IMV with PPi on day 6 than in any other group. Experiments were repeated twice. Values are expressed as the mean percentages ± SEM. Different superscripts a & b in each group of columns denote a significant difference at p<0.05. (D) Excessive concentrations of PPi were added into IVF medium. Fertilization rates decreased with high concentrations of PPi. Experiments were repeated twice. Diagram indicates % monospermic (□) and % polyspermic (▪) fertilization. Values are expressed as the mean percentages of total fertilization ± SEM. Numbers of inseminated ova are indicated in parentheses. (E) Effect of PPi on fertilization with spermatozoa stored in commercial extender, BTS-IMV. Nearly 100% fertilization was achieved using spermatozoa preserved in BTS-IMV with 10 µM PPi (day 3). Diagram indicates % monospermic (□) and % polyspermic (▪) fertilization. Experiments were repeated twice. Values are expressed as the mean percentages of total fertilization ± SEM. Numbers of inseminated ova are indicated in parentheses.
(TIF)

Figure S2 **Immunofluorescence and Western blotting of boar spermatozoa and sperm extracts, respectively, using immunosaturated anti-PPA1 antibody.** Anti-PPA1 antibody (1 mg/ml) was co-incubated with 1 mg/ml recombinant PPA1 protein (1∶20 ratio) at 4°C overnight. Porcine oocytes were fertilized in the presence of anti-PPA1 antibody, immunosaturated anti-PPA1 antibody or non-immune rabbit sera, respectively, after then fixed and stained with GAR-TRITC (red) and DAPI (blue). (A) PPA1 localization (red) in the acrosome of boar spermatozoa by immunofluorescence (a); PPA1 fluorescence was absent after labeling with immunosaturated anti-PPA1 antibody (b) or non-immune rabbit sera (c). (B) Western blotting of the boar sperm-acrosome extract. Lanes 1 and 2 were probed with active anti-PPA1 antibody and immunosaturated anti-PPA1 antibody, respectively. (C) Comparison of IVF in the presence of anti-PPA1 antibody, immunosaturated anti-PPA1 antibody, non-immune rabbit sera or no treatment. Diagram indicates % monospermic (□) and % polyspermic (▪) fertilization. Values are expressed as the mean percentages of total fertilization ± SEM. Different superscripts a, b & c in each histogram denote a significant difference at p<0.05, meaning that column a is significantly different from columns b and c, and column b is significantly different from columns a and c. Numbers of inseminated ova are indicated in parentheses.
(TIF)

Figure S3 **Addition of glucose (100 µM-1 mM) did not contribute to the PPi induced fluorescence of boar seminal plasma (A; 10 µg/ml), porcine oviductal fluid (B; 10 µg/ml), boar spermatozoa (C; 1×10^6^ spermatozoa/ml) and control (D; without spermatozoa).** Experiments were repeated three times. The emitted fluorescence (no units) was measured at multiple time points to follow the kinetics of the reaction (excitation 530 nm; emission 590 nm). Values are expressed as the mean of fluorescence intensity ± SEM.
(TIF)

Figure S4 **Negative control for Western blotting (** **Fig. 4** **).** Membranes with boar, bull and mouse sperm extracts probed with immunosaturated anti-PPA1 antibody and GAR-IgG-HRP. There are no specific bands.
(TIF)

Figure S5 **Immunofluorescence of PPA1 in permeabilized (A; connecting piece and postacrosomal sheath labeling) and non-permeabilized (B; no labeling) boar spermatozoa, mouse spermatozoa (c; acrosome labeling), human spermatozoa (D; connecting piece & midpiece labeling), pig oocyte cumulus complex (E; cumulus cell labeling), zona-enclosed oocyte (F; ooplasm labeling) and zona-free oocyte (G; ooplasm and metaphase II-spindle labeling).**
(TIF)

Figure S6 **Flow cytometric scatter diagrams reflecting the changes in sperm viability and mitochondrial membrane potential, induced by PPi supplementation during semen storage.** Boar spermatozoa were stored in BTS in the presence/absence of 10 µM PPi for 3 or 10 days. Sperm viability (SYBR14, live/PI, dead) and mitopotential (JC-1, live/7-AAD, dead) were measured by flow cytometry. (A) Spermatozoa preserved in BTS for 3 days. (B) Spermatozoa preserved in BTS with PPi for 3 days. (C) Spermatozoa preserved in BTS for 10 days. (D) Spermatozoa preserved in BTS with PPi for 10 days. (E) Vehicle solution, DMSO was added instead of fluorescent dyes as a control.
(TIF)

Figure S7 **Sperm mitochondrial membrane potential during sperm storage with/without PPi.** Percentages of spermatozoa with polarized (live), depolarizing (dying) and depolarized (dead) mitochondrial membranes. Experiments were repeated three times. Values are expressed as the mean percentages ± SEM.
(TIF)

Figure S8 **Effect of PPi supplementation on the ATP content of boar spermatozoa.** Semen was washed twice with PBS at room temperature, collected by centrifugation at 800×*g* for 5 min and adjusted to concentration of 1×10^6^ spermatozoa/ml with PBS. Sperm ATP content was determined using a luciferase reaction kit with or without cell lysis solution according to the manufacturer's protocol (ATPliteTM, Perkin Elmer Inc., Boston, MA). Standards were prepared from ATP standard (Perkin Elmer) using serial dilutions to obtain concentrations of 1×10^−7^, 1×10^−8^, 1×10^−9^, 1×10^−10^, and 1×10^−11^ M. Aliquots of the ATP stock solution were stored at −20°C until use, and standard curve dilutions were prepared for each assay. Bioluminescence was measured with a Synergy 2 multi-mode microplate reader (Biotek, Winooski, VT) after addition of 100 µl sample and 100 µl luciferin-luciferase reagent. The 96-well plate was incubated at 37.5°C for 30 min, and luminescence was measured at multiple time points to follow the kinetics of the reaction. Sperm motility was examined before and after measurement by light microscopy to confirm that the luciferase reagent did not cause sperm damage. Total ATP (A) or sperm-surface ATP (ssATP) (B) content of fresh semen was measured by with/without lysis solution. Total ATP (C) or ssATP (D) contents of spermatozoa preserved for 3–5 days was measured with/without lysis solution. Experiments were repeated three times. Values are expressed as the mean ± SEM. Different superscripts a & b in each histogram denote a significant difference at p<0.05, meaning that column a is significantly different from column b, and column ab is not significantly different from either a or b.
(TIF)
