# Supplementary figures and images for: Identification of the Inorganic Pyrophosphate Metabolizing, ATP Substituting Pathway in Mammalian Spermatozoa
Source: PLoS One. 2012 Apr 2;7(4):e34524. doi: 10.1371/journal.pone.0034524 (PMC3317647; doi:10.1371/journal.pone.0034524)

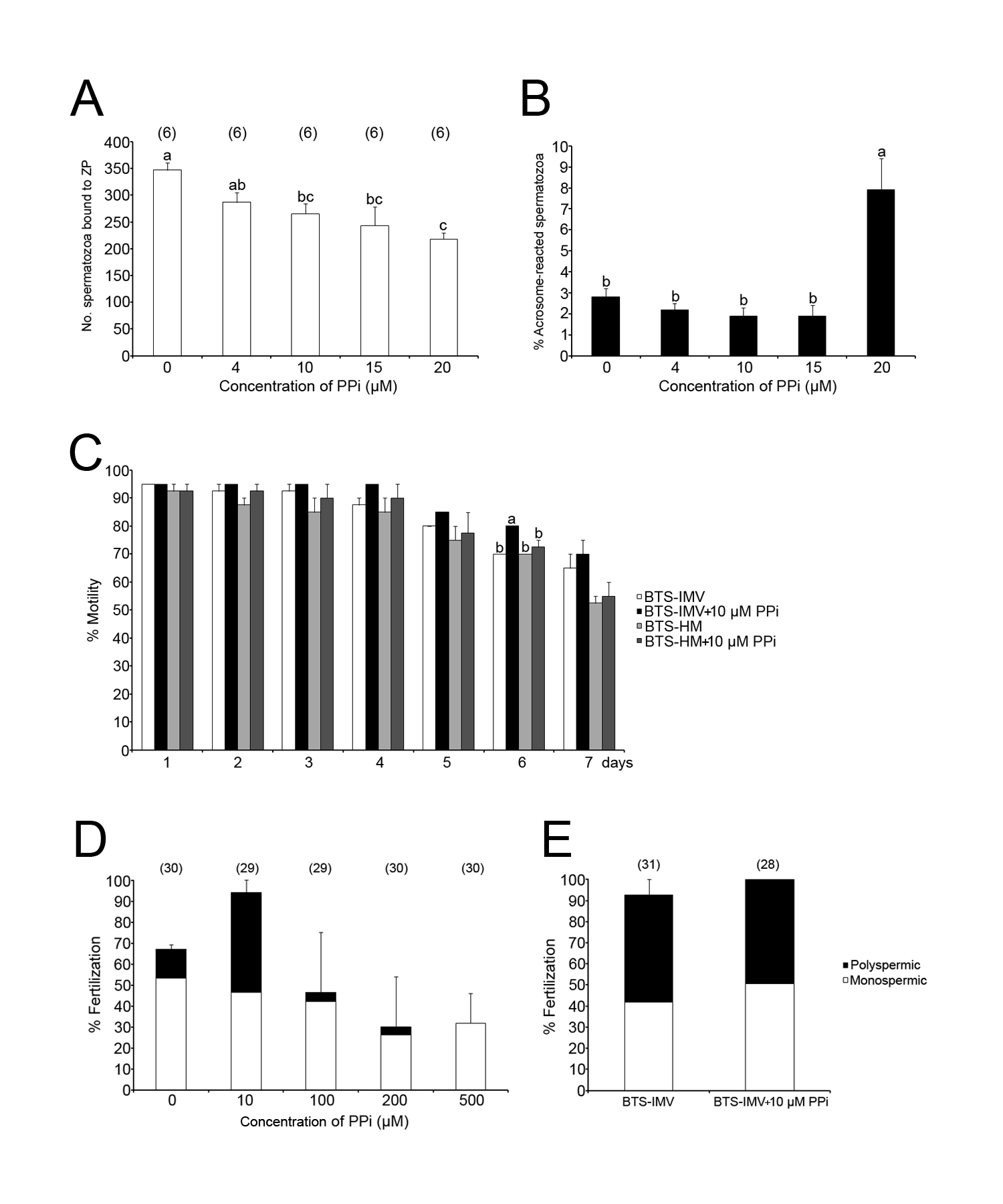

Supplement: Figure S1 — (A) Effect of PPi on sperm-zona binding. Porcine oocytes were inseminated (sperm conc. 5×10^5^ spermatozoa/ml) with various concentrations of PPi for 30 min, fixed and stained with DNA stain DAPI. The numbers of spermatozoa bound per zona-pellucida (ZP) were counted under epifluorescence microscope. Values are expressed as the mean number ± SEM. Different superscripts a, b & c in each histogram denote a significant difference at p<0.05, meaning that column a is significantly different from columns b and c, column b is significantly different from columns a and c, column ab is not significantly different from either a or b, and column bc is not significantly different from columns b and c. Numbers of inseminated ova are indicated in parentheses. (B) The percentage of acrosome-reacted spermatozoa of panel A (PNA-FITC stained). Values are expressed as the mean percentages ± SEM. Different superscripts a & b in each group of columns denote a significant difference at p<0.05. (C) Effect of PPi supplementation on the viability of spermatozoa stored in commercial and custom made BTS extenders. Boar spermatozoa were preserved in BTS-IMV (IMV technologies, France) or BTS-HM (homemade) with/without 10 µM PPi for 7 days at room temperature. The percentage of motile spermatozoa was estimated at 38.5°C using a light microscope at 250× magnification. Higher sperm motility was observed in BTS-IMV with PPi on day 6 than in any other group. Experiments were repeated twice. Values are expressed as the mean percentages ± SEM. Different superscripts a & b in each group of columns denote a significant difference at p<0.05. (D) Excessive concentrations of PPi were added into IVF medium. Fertilization rates decreased with high concentrations of PPi. Experiments were repeated twice. Diagram indicates % monospermic (□) and % polyspermic (▪) fertilization. Values are expressed as the mean percentages of total fertilization ± SEM. Numbers of inseminated ova are indicated in parentheses. (E) Eff [file pone.0034524.s001.tif]

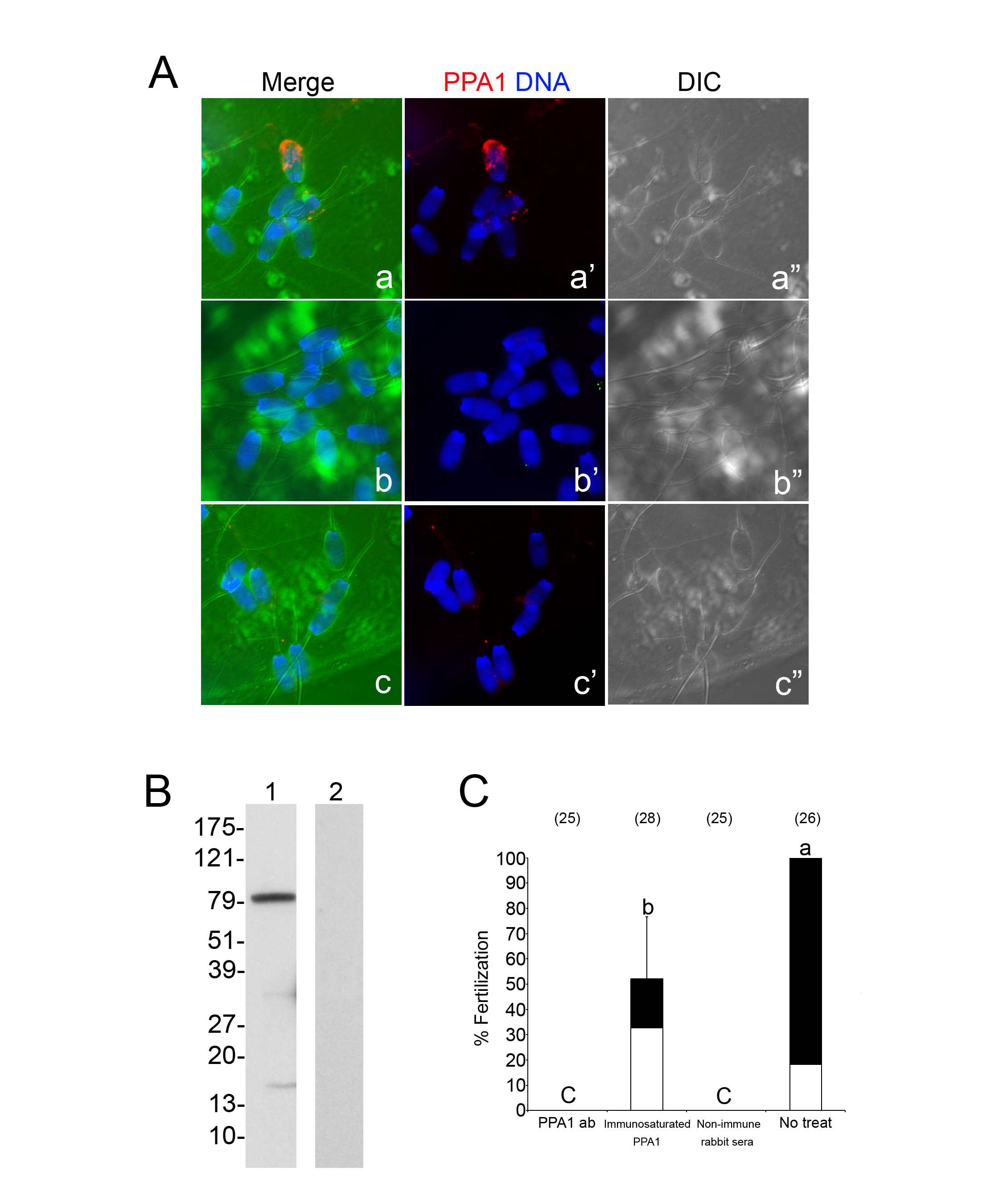

Supplement: Figure S2 — Immunofluorescence and Western blotting of boar spermatozoa and sperm extracts, respectively, using immunosaturated anti-PPA1 antibody. Anti-PPA1 antibody (1 mg/ml) was co-incubated with 1 mg/ml recombinant PPA1 protein (1∶20 ratio) at 4°C overnight. Porcine oocytes were fertilized in the presence of anti-PPA1 antibody, immunosaturated anti-PPA1 antibody or non-immune rabbit sera, respectively, after then fixed and stained with GAR-TRITC (red) and DAPI (blue). (A) PPA1 localization (red) in the acrosome of boar spermatozoa by immunofluorescence (a); PPA1 fluorescence was absent after labeling with immunosaturated anti-PPA1 antibody (b) or non-immune rabbit sera (c). (B) Western blotting of the boar sperm-acrosome extract. Lanes 1 and 2 were probed with active anti-PPA1 antibody and immunosaturated anti-PPA1 antibody, respectively. (C) Comparison of IVF in the presence of anti-PPA1 antibody, immunosaturated anti-PPA1 antibody, non-immune rabbit sera or no treatment. Diagram indicates % monospermic (□) and % polyspermic (▪) fertilization. Values are expressed as the mean percentages of total fertilization ± SEM. Different superscripts a, b & c in each histogram denote a significant difference at p<0.05, meaning that column a is significantly different from columns b and c, and column b is significantly different from columns a and c. Numbers of inseminated ova are indicated in parentheses. (TIF) [file pone.0034524.s002.tif]

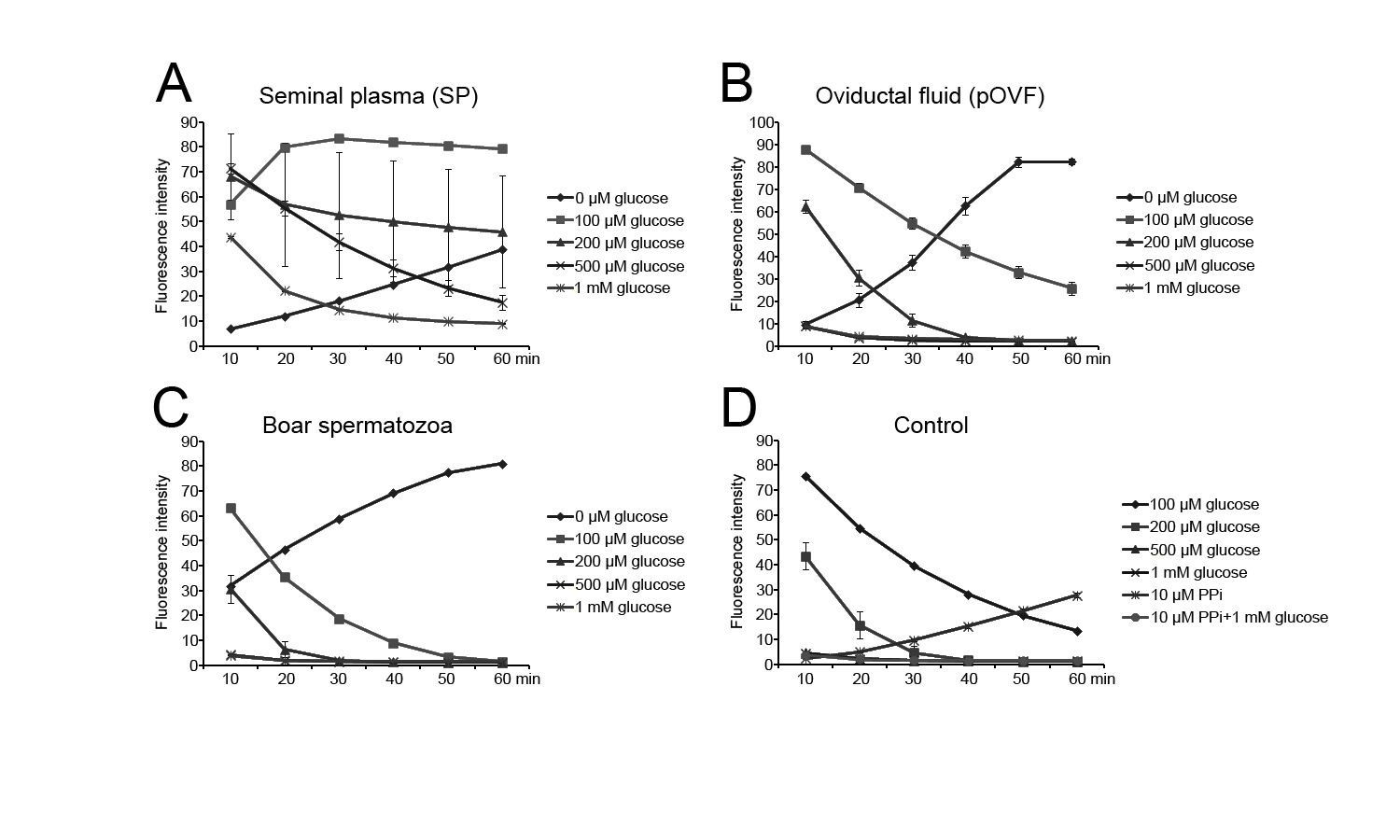

Supplement: Figure S3 — Addition of glucose (100 µM-1 mM) did not contribute to the PPi induced fluorescence of boar seminal plasma (A; 10 µg/ml), porcine oviductal fluid (B; 10 µg/ml), boar spermatozoa (C; 1×10^6^ spermatozoa/ml) and control (D; without spermatozoa). Experiments were repeated three times. The emitted fluorescence (no units) was measured at multiple time points to follow the kinetics of the reaction (excitation 530 nm; emission 590 nm). Values are expressed as the mean of fluorescence intensity ± SEM. (TIF) [file pone.0034524.s003.tif]

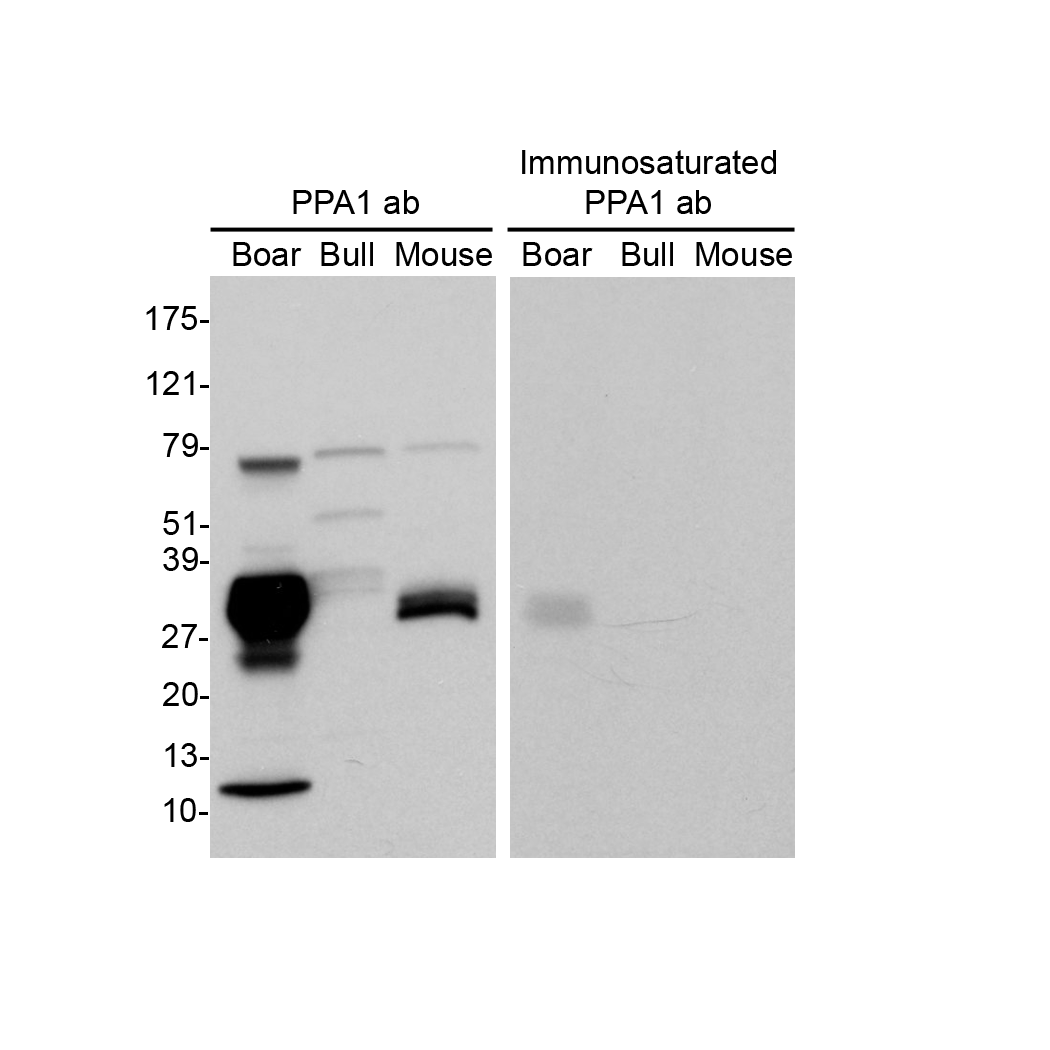

Supplement: Figure S4 — Negative control for Western blotting ( Fig. 4 ). Membranes with boar, bull and mouse sperm extracts probed with immunosaturated anti-PPA1 antibody and GAR-IgG-HRP. There are no specific bands. (TIF) [file pone.0034524.s004.tif]

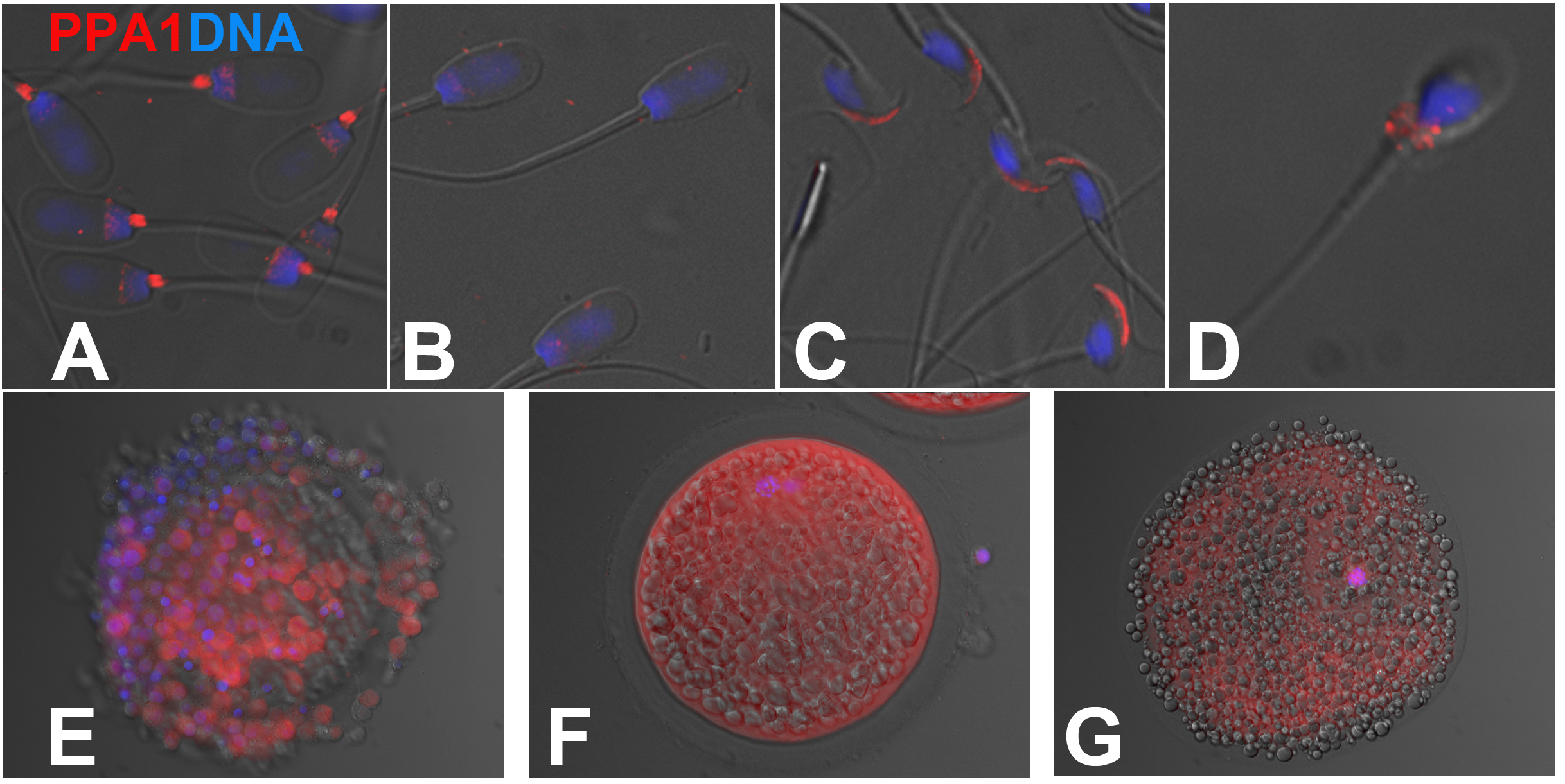

Supplement: Figure S5 — Immunofluorescence of PPA1 in permeabilized (A; connecting piece and postacrosomal sheath labeling) and non-permeabilized (B; no labeling) boar spermatozoa, mouse spermatozoa (c; acrosome labeling), human spermatozoa (D; connecting piece & midpiece labeling), pig oocyte cumulus complex (E; cumulus cell labeling), zona-enclosed oocyte (F; ooplasm labeling) and zona-free oocyte (G; ooplasm and metaphase II-spindle labeling). (TIF) [file pone.0034524.s005.tif]

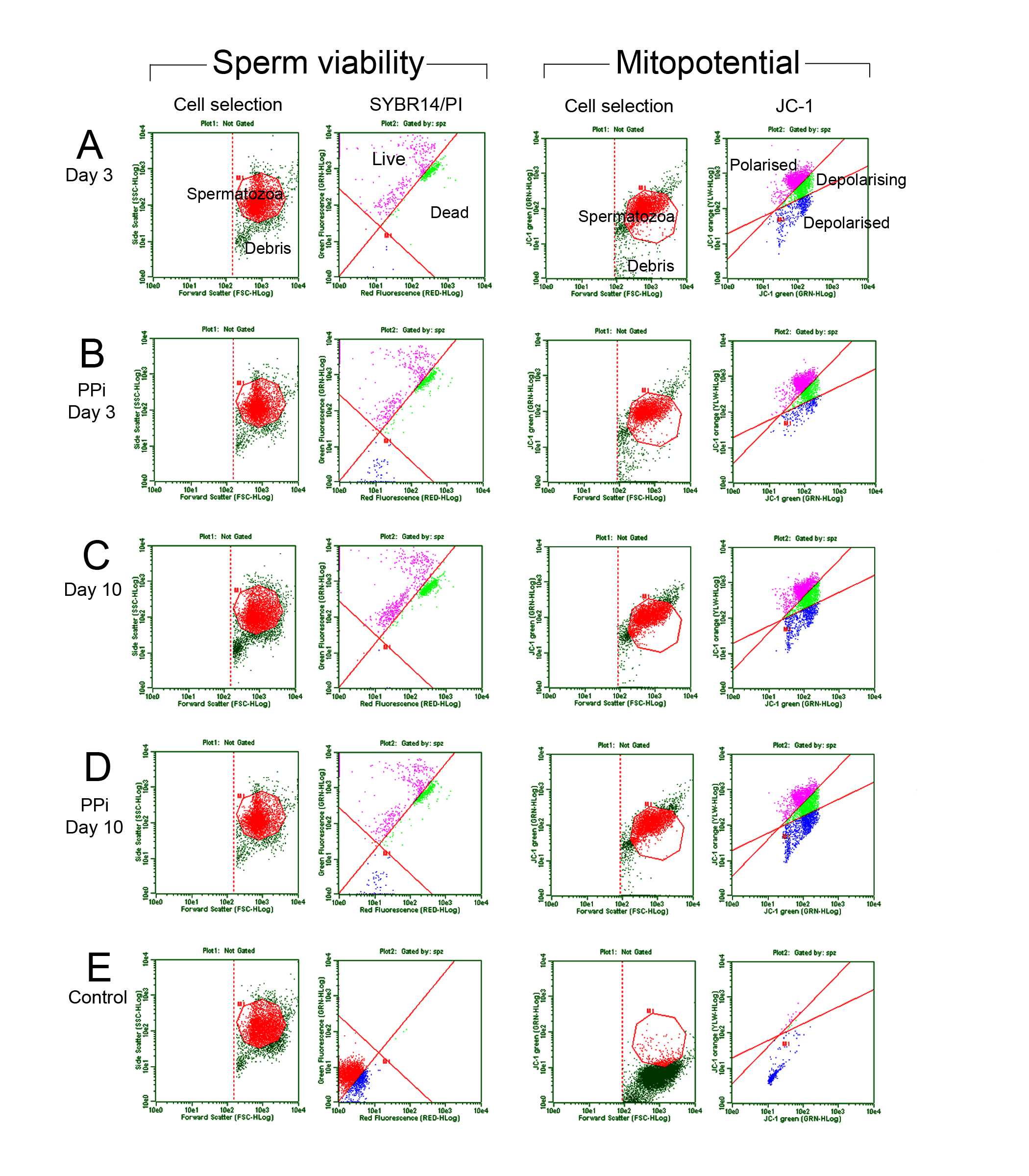

Supplement: Figure S6 — Flow cytometric scatter diagrams reflecting the changes in sperm viability and mitochondrial membrane potential, induced by PPi supplementation during semen storage. Boar spermatozoa were stored in BTS in the presence/absence of 10 µM PPi for 3 or 10 days. Sperm viability (SYBR14, live/PI, dead) and mitopotential (JC-1, live/7-AAD, dead) were measured by flow cytometry. (A) Spermatozoa preserved in BTS for 3 days. (B) Spermatozoa preserved in BTS with PPi for 3 days. (C) Spermatozoa preserved in BTS for 10 days. (D) Spermatozoa preserved in BTS with PPi for 10 days. (E) Vehicle solution, DMSO was added instead of fluorescent dyes as a control. (TIF) [file pone.0034524.s006.tif]

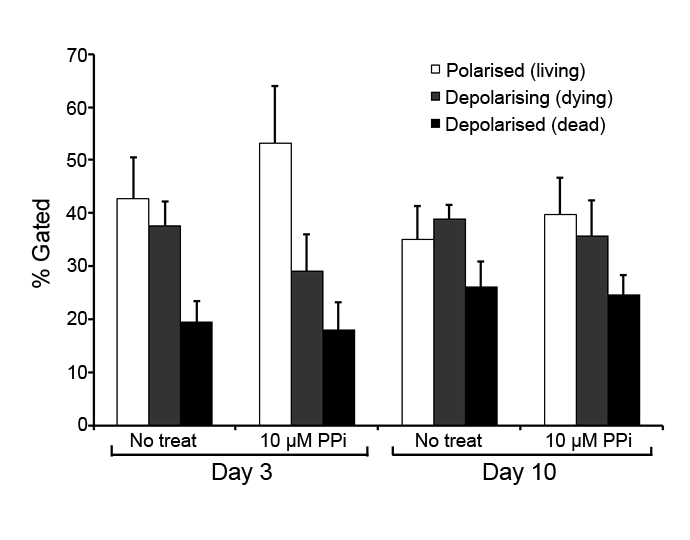

Supplement: Figure S7 — Sperm mitochondrial membrane potential during sperm storage with/without PPi. Percentages of spermatozoa with polarized (live), depolarizing (dying) and depolarized (dead) mitochondrial membranes. Experiments were repeated three times. Values are expressed as the mean percentages ± SEM. (TIF) [file pone.0034524.s007.tif]

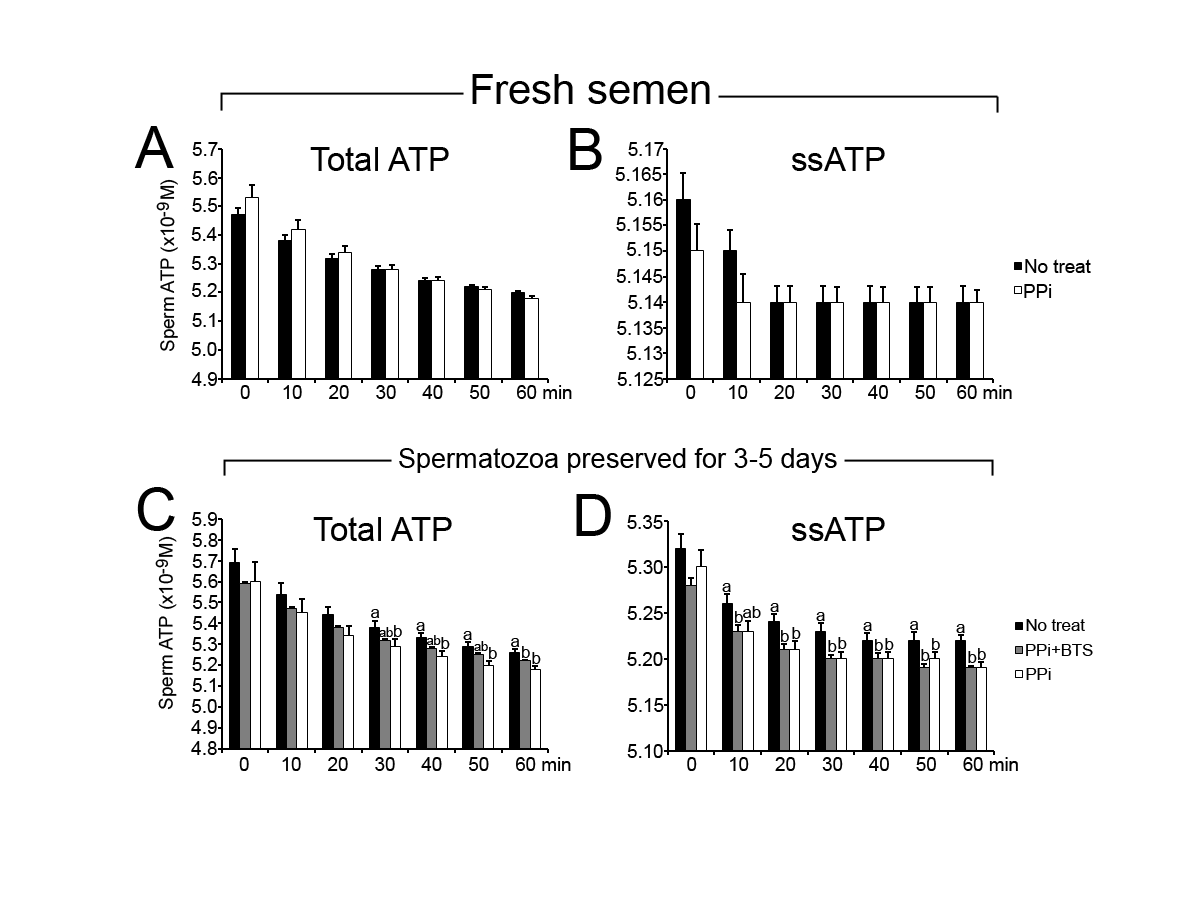

Supplement: Figure S8 — Effect of PPi supplementation on the ATP content of boar spermatozoa. Semen was washed twice with PBS at room temperature, collected by centrifugation at 800×g for 5 min and adjusted to concentration of 1×10^6^ spermatozoa/ml with PBS. Sperm ATP content was determined using a luciferase reaction kit with or without cell lysis solution according to the manufacturer's protocol (ATPliteTM, Perkin Elmer Inc., Boston, MA). Standards were prepared from ATP standard (Perkin Elmer) using serial dilutions to obtain concentrations of 1×10^−7^, 1×10^−8^, 1×10^−9^, 1×10^−10^, and 1×10^−11^ M. Aliquots of the ATP stock solution were stored at −20°C until use, and standard curve dilutions were prepared for each assay. Bioluminescence was measured with a Synergy 2 multi-mode microplate reader (Biotek, Winooski, VT) after addition of 100 µl sample and 100 µl luciferin-luciferase reagent. The 96-well plate was incubated at 37.5°C for 30 min, and luminescence was measured at multiple time points to follow the kinetics of the reaction. Sperm motility was examined before and after measurement by light microscopy to confirm that the luciferase reagent did not cause sperm damage. Total ATP (A) or sperm-surface ATP (ssATP) (B) content of fresh semen was measured by with/without lysis solution. Total ATP (C) or ssATP (D) contents of spermatozoa preserved for 3–5 days was measured with/without lysis solution. Experiments were repeated three times. Values are expressed as the mean ± SEM. Different superscripts a & b in each histogram denote a significant difference at p<0.05, meaning that column a is significantly different from column b, and column ab is not significantly different from either a or b. (TIF) [file pone.0034524.s008.tif]
